# A new method for near real-time, nationwide surveillance of nosocomial COVID-19 in Norway: providing data at all levels of the healthcare system, March 2020 to March 2022

**DOI:** 10.2807/1560-7917.ES.2023.28.12.2200493

**Published:** 2023-03-23

**Authors:** Håvard Skagseth, Silje Bakken Jørgensen, Jacqui Reilly, Oliver Kacelnik

**Affiliations:** 1 Norwegian Institute of Public Health, Department of Infection Prevention and Preparedness, Oslo, Norway; 2 Akershus University Hospital, Oslo, Norway; 3 Safeguarding Health through Infection Prevention Research Group, Research Centre for Health, Glasgow Caledonian University, Glasgow, United Kingdom

**Keywords:** healthcare-associated infection, nosocomial infection, real-time automated surveillance, health register, COVID-19, SARS-CoV-2

## Abstract

**Background:**

Great efforts have been made to minimise spread and prevent outbreaks of COVID-19 in hospitals. However, there is uncertainty in identifying nosocomial vs community-acquired infections. To minimise risks and evaluate measures, timely data on infection risk in healthcare institutions are required.

**Aims:**

To design an automated nationwide surveillance system for nosocomial COVID-19 using existing data to analyse the impact of community infection rates on nosocomial infections, to explore how changes in case definitions influence incidence and to identify patients and wards at highest risk and effects of SARS-CoV-2 variants.

**Methods:**

We used data from the Norwegian real-time emergency preparedness register (Beredt C19), which includes all patients nationwide admitted to Norwegian hospitals between March 2020 and March 2022 with a positive SARS-CoV-2 PCR test during their hospital stay or within 7 days post-discharge. COVID-19 cases were assigned to categories depending on the time between admission and testing.

**Results:**

Infection rates for definite/probable nosocomial COVID-19 increased from 0.081% in year 1 to 0.50% in year 2 in hospital admissions 7 days or longer. Varying the definitions resulted in large changes in registered nosocomial infections. Infection rates were similar across different ward types. By 2022, 58% of patients with a definite/probable nosocomial infection had received three vaccine doses.

**Conclusion:**

Automated national surveillance for nosocomial COVID-19 is possible based on existing data sources. Beredt C19 provided detailed information with only 5% missing data on hospitals/wards. Epidemiological definitions are possible to standardise, enabling easier comparison between regions and countries.

**Key public health message**

**What did you want to address in this study?**

Few surveillance systems capture nosocomial COVID-19 in an easy and timely manner. We wished to establish a national surveillance system for healthcare-associated COVID-19 that provides timely data at national, regional, hospital and ward level, including the number of admissions, infections, and vaccine status. We also wanted to identify people and wards at higher risk, and if the information provided would influence national policy.

**What have we learnt from this study?**

Using existing information sources, we merged data to provide timely information on healthcare-associated infections of COVID-19. Small variations in definitions greatly impacted the number of infections. We identified wards in 95% of cases. The number of healthcare-associated infections increased over the course of the pandemic and more infections arose in unvaccinated individuals.

**What are the implications of your findings for public health?**

In anticipation of further COVID-19 waves and winter respiratory virus seasons, hospitals and national authorities will need updated and quality-assured data on healthcare-associated respiratory infections. Automated surveillance using existing databases provides quality data down to the level of the individual without increasing the workload for frontline caregivers. It will be important to agree on definitions for nosocomial COVID-19 internationally.

## Introduction

During the coronavirus disease (COVID-19) pandemic, great efforts have been made to prevent outbreaks and minimise secondary spread in hospitals. Infection control is of vital importance for patient safety and preserving the function of healthcare services. To this end, hospitals have focused on improving the implementation of their transmission-based precautions and following the hierarchy of controls to reduce introduction and intra-hospital spread of the severe acute respiratory syndrome coronavirus 2 (SARS-CoV-2).

Implementation of measures for infection control should consider not only their benefits but also their impact on day-to-day activities. To maintain proportionality and target infection prevention measures appropriately, we need to understand transmission in hospitals. However, defining nosocomial COVID-19 is difficult because of variable incubation time and uncertainty about where the patient was infected (hospital or elsewhere). Recording nosocomial infections locally is labour-intensive [1] and few surveillance systems are designed for this purpose.

The Norwegian Institute of Public Health (NIPH) and the Norwegian Directorate of Health established a new real-time preparedness register ‘Beredt C19’, a database made up of separate modules to aid the pandemic response [2]. This provided the NIPH with the opportunity to test a new method for national surveillance of epidemiologically defined nosocomial COVID-19 by merging data from diverse national registers with individual-level data.

Surveillance of nosocomial infections is moving from manual data collection by clinical staff to greater reliance on electronic systems and new automated and semi-automated techniques such as data mining of patient records [3]. Given the workload of front-line caregivers, it is crucial to explore more automated methods of collecting the data at both the national and ward level. There is no established definition for the parameters that define a nosocomial COVID-19 infection for surveillance purposes, and several different definitions have been used [4]. 

Our aim was to establish a simple system that could provide information at all levels of the health system in near real-time. The system should be robust but flexible enough to adapt quickly to changes such as altered incubation time, emergence of new SARS-CoV-2 variants or vaccination coverage. Here, the objectives were: (i) to use existing data on hospital admissions, vaccination status and our notifiable disease database to design a nationwide surveillance system for nosocomial COVID-19 and to specifically test whether we could capture data on hospital stays, wards, vaccine status and demographics and determine the level of completeness, (ii) to investigate how changing the epidemiological definition of nosocomial COVID-19 affects the incidence rates and (iii) to identify high risk patients and hospital wards and to consider the effects of SARS-CoV-2 variant type and community transmission on the rates of nosocomial COVID-19.

## Methods

### Data Sources

All data for analysis were extracted from the Norwegian preparedness register, Beredt C19, as described elsewhere [2]. To develop the system, we extracted and merged data from the following component registers: (i) the Norwegian Surveillance System for Communicable Diseases (MSIS), which contains all positive SARS-CoV-2 PCR tests from Norwegian laboratories (which also includes some rapid antigen tests towards the end of the study period), (ii) the Norwegian Patient Register (NPR) data on all hospital stays in Norway and (iii) the Norwegian Immunisation Register (SYSVAK) that contains all vaccine doses registered in Norway. MSIS, NPR and SYSVAK data are updated daily.

For data protection purposes, each surveillance group in Beredt C19 is responsible for one surveillance question and only has access to data needed to carry out their responsibilities. Merging of the individual registers was conducted using national person identifiers, which were not visible to data analysts.

Data elaboration and analyses were done using R version 4.0.2 [5].

### Surveillance method, case definition and population at risk

Using NPR [6], all individuals with an overnight hospital stay in Norway between week 10 2020–week 10 2022 were included. The study period was divided into 2 years (year 1: week 10 2020–week 9 2021; year 2: week 10 2021–week 10 2022). Cases were defined as people who had a positive SARS-CoV-2 PCR test during their hospital stay or within 7 days after discharge. Hospital stays that were 1 day or less apart were combined as a single admission, retaining the ward and hospital data for the first stay only. In most instances, this was an artefact of the register representing people transferred between wards in the same hospital. In a few cases, this was time spent outside hospital leading to potential confounding. However, this was considered more accurate than treating stays on different wards as separate admissions. For patients admitted to hospital during the last week of each year with no discharge date (due to systematic errors), it is assumed that they were discharged 1 January.

Nosocomial SARS-CoV-2 infections were initially assigned to four categories based on the time interval between admission, discharge and positive test: Community-acquired infection (CAI), indeterminate, probable, or definite healthcare-associated infection (HAI). The initial definition was based on one implemented by National Health Service (NHS) Scotland [7] (described below) with a few modifications. We tested how different definition criteria, i.e. different estimates of incubation time, influenced case distribution through a form of sensitivity analysis. For anyone with two separate stays (2 or more days apart) and/or multiple positive tests, we kept the stay with the highest probability of being nosocomial (see below). In an ongoing surveillance system, both stays could be included for those having two positive tests, but for the period we were examining, this does not impact our results.

To classify which part of the hospital system the patient was located, we used the codes from the NPR based on the department where they spent their first night during the admission. Five different categories were constructed: surgery, internal medicine, obstetrics/gynaecology, orthopaedics and paediatrics. The risk ratio was calculated between the categories of wards to determine if there was a significant difference between them. For this study, all admissions to psychiatric hospitals and drug-dependency units were excluded. The number of nosocomial COVID-19 cases for psychiatry and drug-dependency can be found in the Supplementary Figures S1 and S2.

Vaccine status was assigned using information from the SYSVAK. We used the vaccination status at the start of the hospital stay related to their positive test [8].

### Definitions of nosocomial COVID-19

**Figure 1:**
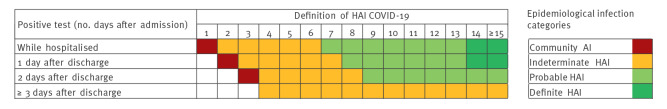
Classification of nosocomial COVID-19 type based on timing of positive test after hospital admission, Norway

AI: acquired infection; COVID-19: coronavirus disease; HAI: healthcare-associated infection.

The day of admission was counted as day 0 (Figure 1). All cases with a positive PCR test for SARS-CoV-2 within 1-day post-admission were classified as CAI. This was to ensure that everyone who tested positive after admission was considered and evaluated whether they were infected in the hospital as per the definition. If the definitions changed, they could be defined differently. A ‘definite HAI’ defined cases who had a positive test while hospitalised if the sample was taken 14 days or more after admission and those who tested positive on their first day post-discharge if they had been admitted to hospital 14 or more days before. A ‘probable HAI*’* defined cases who (i) had a positive test 7–13 days post-admission, or (ii) tested positive on their first day post-discharge if they had been admitted to hospital 8–13 days before and (iii) those who tested positive 9 or more days post-admission on day 2 post-discharge. An ‘indeterminate HAI’ included all those who either had (i) a positive test 2–6 days after admission and were still in the hospital, (ii) a positive test 1 day after discharge and 3–7 days after admission, (iii) a positive test 2 days after discharge and 4–8 days after admission or (iv) a positive test 3–7 days after discharge.

In the sensitivity analysis, the number of days of hospitalisation needed was varied for the different definitions. The minimum hospital stay was varied from 15 to 8 days for definite HAIs and the lower limit for probable HAIs between 12 and 5 days. Definition 9 (Table 1) corresponds to the surveillance definition adopted by both the United Kingdom NHS and the United States Centers for Disease Control and Prevention (CDC) and is the definition used for all other analyses in this article [4].

**Table 1:** Definitions of nosocomial COVID-19 tested in the sensitivity analysis, Norway, March 2020−March 2022

Definition	Community AI	Indeterminate HAI	Probable HAI	Definite HAI
1	1 day	2–11 days	12–14 days	≥ 15 days
2	1 day	2–10 days	11–13 days	≥ 14 days
3	1 day	2–9 days	10–12 days	≥ 13 days
4	1 day	2–8 days	9–11 days	≥ 12 days
5	1 day	2–7 days	8–10 days	≥ 11 days
6	1 day	2–6 days	7–9 days	≥ 10 days
7	1 day	2–5 days	6–8 days	≥ 9 days
8	1 day	2–4 days	5–7 days	≥ 8 days
9	1 day	2–6 days	7–13 days	≥ 14 days

SARS-CoV-2: severe acute respiratory syndrome coronavirus 2; AI: acquired infection; HAI: healthcare-associated infection.

Days provided indicate day(s) during which positive test occurred after hospital admission.

### Comparative analysis

For further analysis, Definition 9 was used to compare rates of HAI against the community infection rate at any given time during the study period. To visualise the correspondence and given the difference in scale, HAI was plotted against 0.1% of the community rate. This enabled visualisation of the pattern on the same scale. This was repeated in four pre-determined age groups (0–19, 20–39, 40–59, ≥ 60 years) to examine the effect of age on this distribution.

## Results

### Designing a nationwide surveillance system

From NPR data, there were 955,598 patients who had stays registered as overnight hospital admissions during the study period, translating to 1,858,515 unique stays, or 6,048,156 patient-days. Applying the algorithm for combining stays and removing day admissions, this translated to 891,410 different people, 1,433,178 stays, or 6,069,919 patient-days. This matches the official figures that Statistics Norway [9] published for 2021, based on NPR (< 1% difference).

Table 2 describes the population in terms of definite, probable or indeterminate HAI in somatic (acute care) hospitals, comparing age and sex distribution to the whole population of patients admitted on the same wards. The median age of cases was 69 years (interquartile range (IQR): 49–80) in year 1 and 59 years (IQR: 34–77) in year 2 and the proportion of women was 45.1% in year 1 and 51.0% in year 2. For patients with probable and definite HAI only, the median age was 75 years (IQR: 57–80) in year 1 and 69 years (IQR: 52–79) in year 2 while the proportion of women was 43.0% in year 1 and 40.5% in year 2. The median age was around 60 years for hospital stays of at least 1 day and around 70 years for hospital stays lasting 7 or more days. This did not change considerably over the 2 years. The proportion of hospitalisations of women was higher than the proportion of women who acquired a HAI.

**Table 2:** Description of the population admitted to hospitals for 1 or more days and 7 or more days each year with nosocomial COVID-19, Norway, March 2020–March 2022 (n = 1,433,687 stays)

Characteristics	Length of hospital stays				HAI			
	1 day or more		7 days or more		Indeterminate, probable, and definite HAI		Probable and definite HAI	
Total	1,433,687 stays		219,666 stays		2,692 cases		600 cases	
Year 1								
Stays/cases, n (%)	691,244	100	105,817	100	288	100	86	100
Median age in years (IQR)	59	32–75	69	52–79	69	49–80	75	57–80
Women, n (%)	371,213	53.7	50,503	47.8	130	45.1	37	43.0
Men, n (%)	320,031	46.3	55,314	52.2	158	54.9	49	57.0
Median length of stay (IQR)	2	1–5	10	8–15	7	3–18	24	15–32
Year 2								
Stays/cases, n (%)	742,443	100	113,849	100	2,404	100	514	100
Median age in years (IQR)	60	32–76	70	52–79	59	34–77	69	52–79
Women, n (%)	399,591	53.8	55,067	48.1	1,226	51.0	208	40.5
Men, n (%)	342,852	46.2	58,782	51.9	1,178	49.0	306	59.5
Median length of stay (IQR)	2	1–5	10	8–15	5	2–12	22	14–40

IQR: interquartile range; HAI: healthcare-associated infection.

Results for year 1 (week 10 2020–week 9 2021) are represented in the first five rows and for year 2 (week 10 2021–week 10 2022) in the last five rows of the table. Infections are as defined in the Methods section (Figure 1).

The COVID-19 patients with probable and definite HAI were considerably younger in year 2 than year 1, while the age distribution for the whole population remained stable. In year 1, there were few HAI cases, and the numbers were driven by a few large outbreaks in wards where older people were infected (data not shown). In year 2, there were more cases and they were distributed over different types of wards and not because of a few outbreaks (data not shown). Of a total of 2,692 potential HAIs during the study period, 57 (2.1%) of the tests were not registered as SARS-CoV-2 PCR tests in MSIS; 52 of the 57 were from 2022.

When stays that were listed in NPR were counted as separate stays and stays that were 1 or less calendar days apart not combined, the total number of probable and definite HAI decreased from 600 to 487.

Through Beredt C19, COVID-19 vaccination status (number of doses) was assigned to the whole population, including cases. Among all people hospitalised for 7 days or more in 2022, 66% had received a third dose at the time they were admitted. Among those who acquired a probable or definite HAI, 58% had received a third dose. At the start of 2022, the SARS-CoV-2 Omicron variant (Phylogenetic Assignment of Named Global Outbreak (Pango) lineage designation B.1.1.529) was dominant in Norway. Information on vaccine coverage in the general population, amongst inpatients and amongst those who developed a HAI can be found in Supplementary Material Table S1.

### Performing sensitivity analysis and investigate changing epidemiological definitions of nosocomial COVID-19

**Figure 2:**
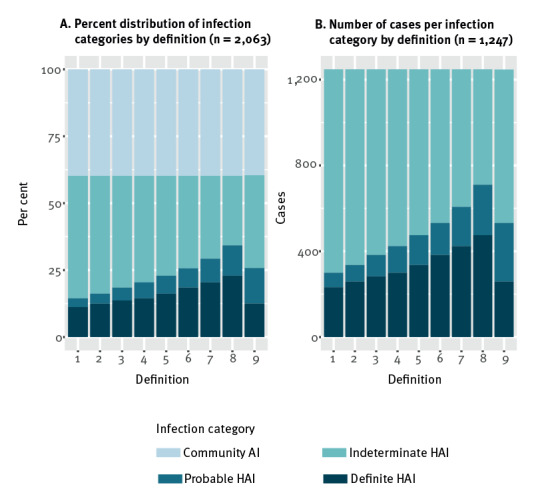
Effects of varying the epidemiological definition to classify COVID-19 patients testing positive during a hospital stay, Norway, March 2020–March 2022

AI: acquired infection; COVID-19: coronavirus disease; HAI: healthcare-associated infection.

In Figure 2, the effects of changing the limits for defining probable HAIs are presented (ranging from 12 days after admission in Definition 1 to 5 days after admission in Definition 8) and definite HAIs (ranging from 15 days after admission in Definition 1 to 8 days in Definition 8), as described in Table 1. To make our results more comparable to other published literature, we included infections that were detected and tested positive for SARS-CoV-2 during the hospital stays, and excluded positive test results after discharge. The results are based on definition 9, as described in the Methods section. The lower limit for probable HAI is then 7 days of hospitalisation and the limit for definite HAI is 14 days of hospitalisation before a definite HAI is considered.

Varying the limit for definite HAI from 15 to 8 days and the lower limit for probable HAI from 12 to 5 days in Definitions 1–8 (Table 1). Results are based on Definition 9, with definite HAI being 14 days and probable HAI being 7–13 days after hospital admission.

### Identifying wards at higher risk of healthcare-associated infections and how infection rates in the community affect hospital infection rates

The hospital ward was identifiable for 95.1% of all stays lasting 7 or more calendar days (Table 3), and for 94.2% of probable and definite HAIs. For the remaining 4.9% and 5.8% respectively, only the hospital trust was identifiable. Most probable and definite HAIs were on internal medicine wards, but those were also the wards with the most stays lasting at least 7 days. There were only small differences in risk between different types of wards, except there were no cases in obstetrics/gynaecology in year 1 and a significantly lower risk for obstetrics/gynaecology wards compared with medicine (Table 4). The category ‘Other’ contains patients initially admitted to emergency rooms, monitoring wards, other specialist wards or smaller hospitals with no organisation by specialty.

**Table 3:** Healthcare-associated infections, and percentage of hospitalisations by ward type, Norway, March 2020–March 2022 (n = 2,692 infections)

Year	Total	Internal Medicine	Obstetrics/ gynaecology	Orthopaedics	Paediatrics	Surgery	Other	Missing
Probable and definite HAI (n)								
1	86	47	0	15	3	17	2	2
2	514	306	14	42	24	78	17	33
Probable, definite and indeterminate HAI (n)								
1	288	135	14	56	10	49	11	13
2	2,404	1,154	318	190	156	388	97	102
Stays lasting 7 or more days (n)								
1	105,510	54,414	6,645	9,723	4,561	19,773	4,264	6,130
2	113,849	60,136	7,517	10,814	5,124	20,937	4,715	4,606
Percentage^a^ of hospitalisations that led to probable or definite HAIs								
1	0.08	0.09	0.00	0.15	0.07	0.09	0.05	0.03
2	0.45	0.51	0.19	0.39	0.47	0.37	0.36	0.71

HAI: healthcare-associated infection.

^a^ Stays starting in year 1 with a positive test in year 2 are classified as stays in year 1 and HAIs in year 2.

**Table 4:** Unadjusted risk ratio for hospital stays ≥ 7 days classified as probable or definite healthcare-associated infections, Norway, March 2020–March 2022 (n = 546 cases)

Ward	Year 1				Year 2			
	Cases	Stays	RR	95% CI	Cases	Stays	RR	95% CI
Internal Medicine	47	54,414	1	Ref.	306	60,136	1	Ref.
Obstetrics/ gynaecology	0	6,645	0.17^a^	0.02–1.24	14	7,517	0.37	0.21–0.63
Orthopaedics	15	9,723	1.85	1.00–3.19	42	10,814	0.76	0.55–1.05
Paediatrics	3	4,561	0.76	0.24–2.45	24	5,124	0.92	0.61–1.39
Surgery	17	19,773	1.00	0.57–1.73	78	20,937	0.73	0.57–0.94

CI: confidence interval; Ref.: reference; RR: risk ratio.

^a^ Using the method of adding 1 to each group to get a RR and 95% CI.

**Figure 3:**
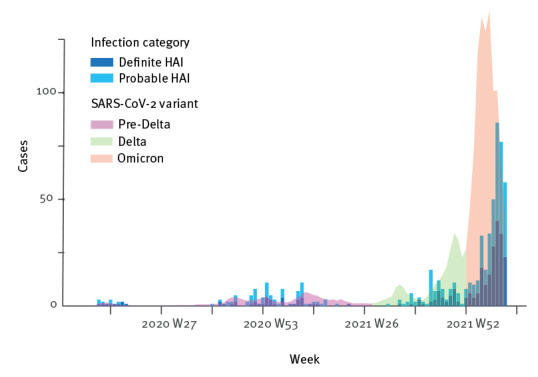
Definite (n = 272) and probable (n = 328) healthcare-associated infections compared to 0.1% of COVID-19 cases in the population, Norway, March 2020–March 2022

**Figure 4:**
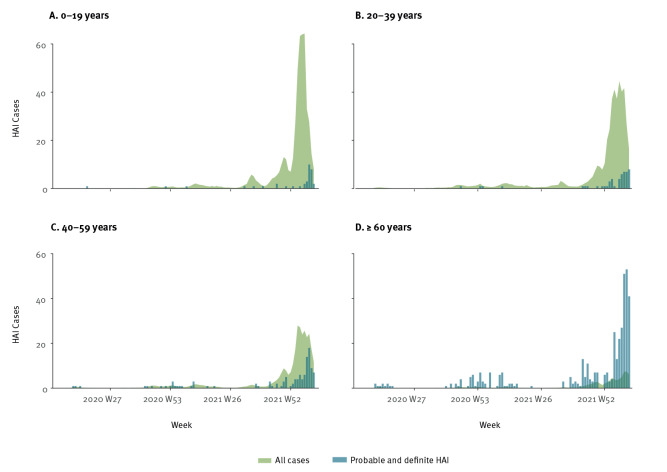
Proportion of COVID-19 cases in the general population compared with healthcare-associated infections by age group, Norway, March 2020–March 2022

The incidence of nosocomial COVID-19 varied over the course of the pandemic. Figure 3 describes the number of probable and definite HAI against 0.1% of all registered cases in relation to the dominant variant in Norway [10,11]. There were few nosocomial infections early in the pandemic, and no cases during the summer 2020 before a new surge during autumn/winter 2020/21. There were few cases summer 2021, before an outbreak with the SARS-CoV-2 Delta (Pango lineage designation B.1.617.2) variant during autumn 2021 peaking at week 43 (October) with 17 probable and definite HAIs. Then, with the introduction of Omicron, the number of HAIs increased during winter 2021/22, peaking at 86 cases in week 8 (February) 2022. Cases were detected at 57 of 83 different hospitals during the study period through NPR and MSIS. In Figure 4, the results are divided by age groups (0–19, 20–39, 40–59 and ≥ 60 years), with 0.1% of registered cases in each age group. Supplementary Figures S1 and S2 show the total number of cases for drug dependency and psychiatry hospitals. Considering proportions of all cases in the population, 0.044% of all registered cases were probable or definite HAI. Dividing this number by age group, the proportion of HAIs for ages 60 years and older was 0.48% of all registered cases, 0.037% for ages 40–59 years, 0.011% for ages 20–39 years and 0.007% for ages 0–19 years.

HAI:  healthcare-associated infection. The dominant SARS-CoV-2 variants are indicated: circulating variants before the emergence of Delta (purple), Delta (green) and Omicron (orange).

HAI: healthcare-associated infections.

The blue bars show the combined number of probable and definite healthcare-associated infections in each age group per week. The green shaded area shows 0.1% of all COVID-19 cases in the population in each age group per week.

## Discussion

Understanding the magnitude of nosocomial COVID-19, other nosocomial infections and hospital function is vital for planning for coming winter respiratory virus seasons. However, identifying cases is difficult and often achieved retrospectively [1,12]. Using de-identified individual level data on all hospital admissions and all positive SARS-CoV-2 PCR-tests taken in Norway, we designed an automated surveillance tool for nosocomial COVID-19 cases. The minimal dataset required to implement accurate surveillance included variables on hospital admissions, discharges and positive test dates. Enhanced surveillance was achieved through the inclusion of variables on the specific hospital, ward type/name and vaccine status.

A retrospective simulation of near-real time surveillance was able to follow the changing incidence of nosocomial infections of COVID-19 in Norwegian hospitals. The system detected cases and was able to classify the ward level in most of the country’s hospitals in a manner that could be reported back within days of positive tests. Furthermore, we have established that, in the future, this could be run prospectively for timely surveillance. During the pandemic, the number of probable and definite HAIs have varied greatly, in large part reflecting the level of community transmission rather than the implementation of specific measures in the hospitals. This finding is comparable to results published by NHS Scotland [7].

The system is based on existing data that is collected for other purposes and requires no extra activity from the health sector. All SARS-CoV-2 PCR test results analysed by a Norwegian laboratory are automatically transferred to our national laboratory database. Combining this information with records on hospital stays from the financial compensation system and vaccine status from our vaccine register (see Supplementary Material) has been effective for giving a picture of infections related to hospital admissions. The changing incubation time of SARS-CoV-2 variants makes strict categorisation difficult, but this can be solved by stratifying data in four categories according to the probability of the cases being nosocomial. Furthermore, hospitals and national bodies should have a low threshold for implementing further infection prevention and control (IPC) measures if the need is suspected.

The results presented are dependent on how the hospital stays that are closely linked in time were combined; every transfer between wards could be considered a new stay. The nature of this automated surveillance means it may not capture every case with full accuracy but should give a good indication of changing trends at both the hospital and national level.

Different types of tests for SARS-CoV-2 infections, e.g. rapid lateral flow antigen tests, have been used in some Norwegian hospitals for screening purposes on admittance, but have not been recommended for diagnostic purposes in hospitals. However, the results of such samples when taken from a healthcare worker are notifiable to the national database through a manual system. We included all positive test results that were registered in our national database. These were predominantly PCR tests, but 2.1% of potential nosocomial infections were not registered as PCR tests and were likely rapid antigen tests.

We were not able to identify hospital and ward for around 5% of hospital stays and probable/definite HAIs. Thus, it is uncertain whether they were somatic hospital stays vs psychiatric or drug-dependency stays. This is partially due to how the data are structured and the fact that the data sets that were available were not intended primarily for this purpose. Any changes in data structure or delay in delivery will also acutely affect the timeliness of our system.

The changes in age and sex distribution are probably due to the low number of cases in the beginning of the pandemic. Outbreaks in a few wards greatly affected the overall distribution. Numbers of nosocomial infections of COVID-19 remained low during the Delta wave (possibly because of high vaccine coverage and more comprehensive IPC measures combined with better adherence). However, the Omicron variant led to far more extensive community spread which was clearly reflected by an increase in cases within the hospitals. The surveillance system was robust enough to work at both high and low levels of spread.

The incidence of nosocomial COVID-19 has varied greatly between countries [12-15]. In Norway, we observed a steady rise in the number of infections included in both probable and definite HAIs with the possible definitions in the sensitivity analysis. The sensitivity analysis demonstrated that these numbers can be influenced by our definition of HAI. Furthermore, while definitions based on incubation time and hospital admission work well for conservative estimates of nosocomial infection rates, it will be important to adjust these to reflect changes in incubation periods of different variants. The results suggest that countries could adopt a common set of definitions for the coming winter seasons to more accurately reflect the effect of the local IPC policy.

Manual surveillance is labour intensive and subject to human error. The PRAISE (Providing a Roadmap for Automated Infection Surveillance in Europe) network points out that while automated surveillance allows IPC staff to focus on other important tasks, hospital-based systems still reproduce the inter-hospital variation in reporting seen in manual surveillance [16]. The network has published a roadmap for implementation of large-scale automated surveillance of healthcare-associated infections. PRAISE suggests that the choice of pathogen for surveillance should be influenced by several factors. These include how common it is, the severity of illness associated with it, the ease of detection, accessibility of data and the possibility of preventative actions. Automated national near real-time surveillance of nosocomial COVID-19 cases based on already accessible register data fulfils these criteria and we consider it an ideal starting point for this type of surveillance.

In the preparedness register, there are data to describe infection rates among employees in each hospital trust, and for the population in particular regions. This information could be combined with results of infection rates in patients to further investigate possible links between regional infection incidence and outbreaks among staff and patients. However, this kind of extensive surveillance raises ethical issues on data privacy and was beyond the scope of this study. The data from individuals outside the hospital is very accurate for the 2 first years of the pandemic when PCR tests were free for all and widely available. As testing recommendations have changed and PCR tests are only used for clinical cases, the surveillance system will no longer provide accurate background data on transmission in the wider community or among healthcare staff.

Norway is not unique in our understanding of the need for information on nosocomial COVID-19. The deliberate choice to define day of admission as day 0 instead of day 1 was because we used number of days hospitalised in most of our analyses. This is important to note when comparing to other studies as most studies use day 1 as the day of admission.

## Conclusion

Automated national surveillance for nosocomial COVID-19 is possible based on existing data sources. Beredt C19 provided detailed information with only 5% missing data on hospitals/wards. Epidemiological definitions are possible to standardise enabling easier comparison between regions and countries. However, the system is not designed to provide information on individuals or single wards and is greatly affected by the incubation period of the disease. Furthermore, the applicability of this type of surveillance system is dependent on both the requisite IT infrastructure and the possibility to anonymously track individuals both in time and throughout their patient journeys. Additional data on cluster and incidents at ward level are required for IPC at the local level. However, the hospital level data enables national surveillance and evaluation. We recommend implementation of automated surveillance where possible.

## Supplementary Data

709000 Supplement
