# Supplementary material for: A new method for near real-time, nationwide surveillance of nosocomial COVID-19 in Norway: providing data at all levels of the healthcare system, March 2020 to March 2022
Source: Euro Surveill. 2023 Mar 23;28(12):2200493. doi: 10.2807/1560-7917.ES.2023.28.12.2200493 (PMC10037665; doi:10.2807/1560-7917.ES.2023.28.12.2200493)
Supplement: Supplement [file 22-00493_SKAGSETH_SUPPLEMENT.pdf]

Supplementary material

This supplementary material is hosted by Eurosurveillance as supporting information alongside the article [*A new method for near real-time, nationwide surveillance of nosocomial COVID-19 in Norway: providing data at all levels of the healthcare system, March 2020 to March 2022]* on behalf of the authors who remain responsible for the accuracy and appropriateness of the content. The same standards for ethics, copyright, attributions and permissions as for the article apply. Supplements are not edited by Eurosurveillance and the journal is not responsible for the maintenance of any links or email addresses provided therein.

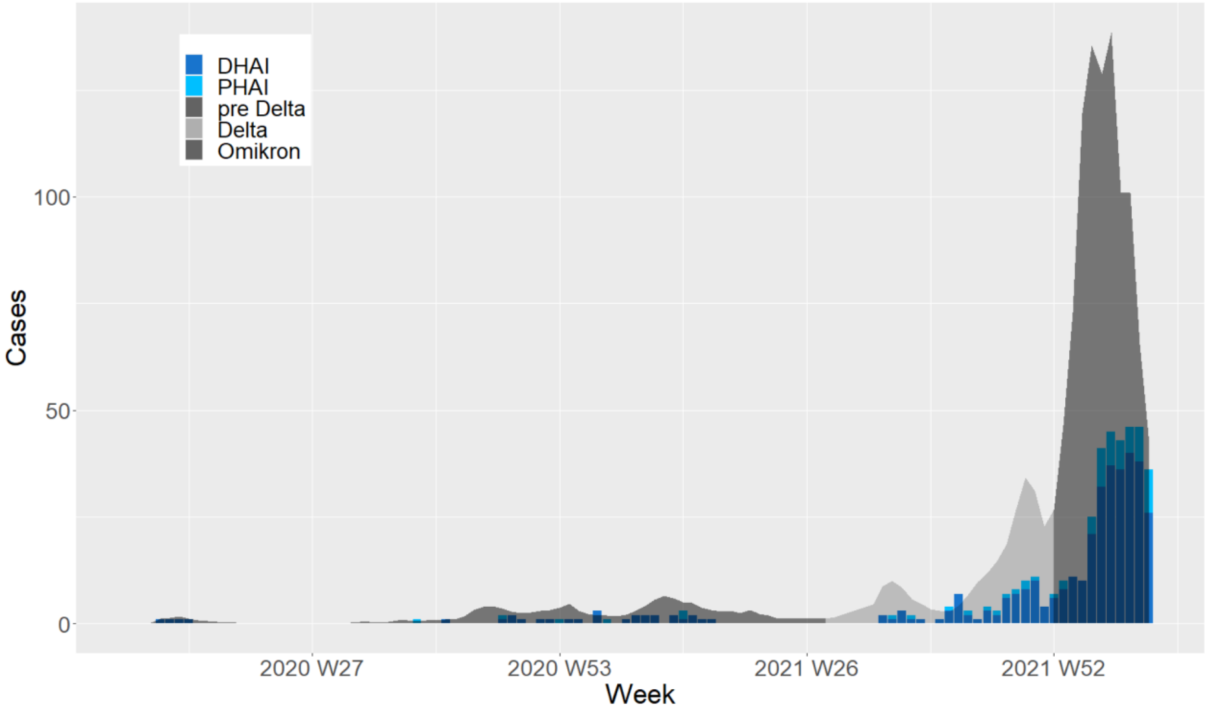

Supplementary figure 1: Cases of definite and probable healthcare-associated infections in drug dependency and psychiatry hospitals with 0.1% of all cases in the population in the background, Norway, March 2020-March 2022. DHAI: Definite HAI; PHAI: Probable HAI; HAI: Healthcare-associated infection; the first grey corresponds to before the delta variant, light grey corresponds to the delta variant being dominant while the second dark grey corresponds to the Omicron variant being dominant.

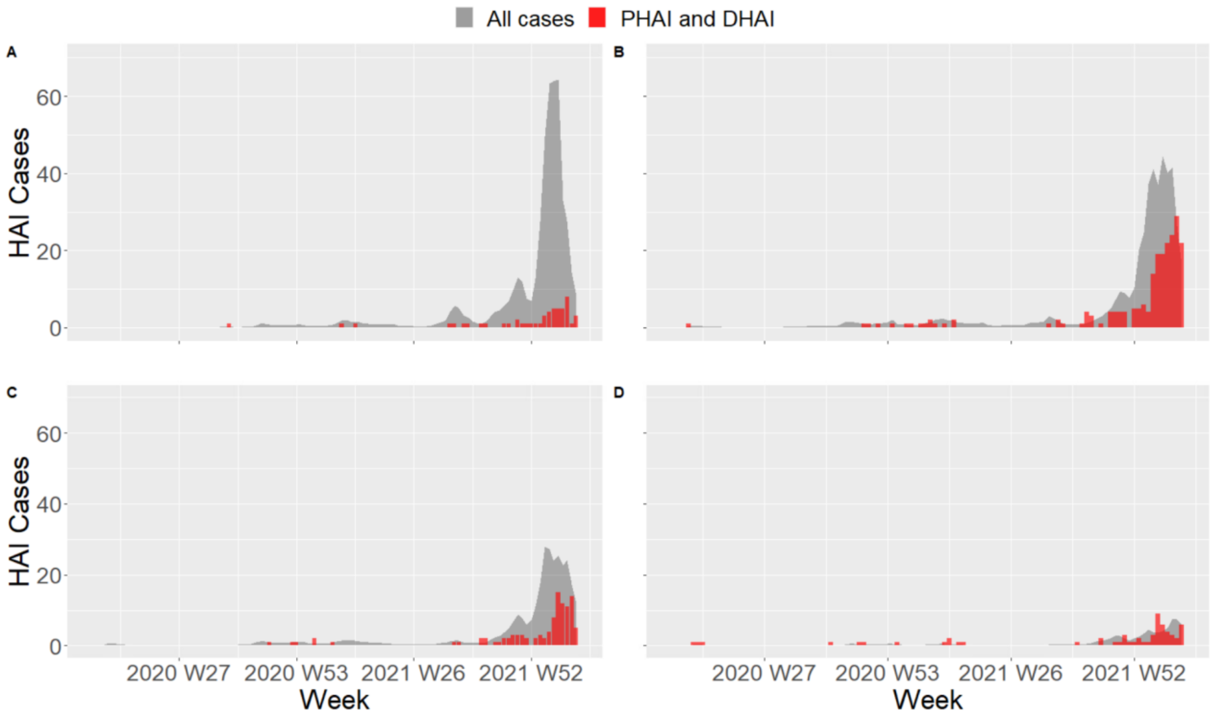

Supplementary figure 2: Cases of definite and probable healthcare-associated infections in drug dependency and psychiatry hospitals with 0.1% of all cases in the population in the background divided into age groups; A: 0-19; B: 20-39; C: 40-59; D: ≥ 60, Norway, March 2020-March 2022; DHAI: Definite HAI; PHAI: Probable HAI; HAI: Healthcare-associated infection;

Supplementary Table 1: Vaccine coverage of the Norwegian population, the people that are hospitalized for seven days or more and the probable healthcare associated infections (probable HAI) and definite healthcare associated infections (definite HAI).

	1 Oct – 31 Dec 2021			1 Jan – 13 Mar 2022		
	% population vaccination coverage (1 Oct/1 Jan)	Admission 7 days or more (n/%)	Probable and definite HAI (n/%)	% population vaccination coverage (1 Jan/13 Mar)	Admission 7 days or more (n/%)	Probable and definite HAI (n/%)
0 or 1 vaccine dose	32/29	5653 / 19	23 / 26	29/27	3572 / 16	80 / 21
2 vaccine doses	68/42	16890 / 58	58 / 67	42/20	4177 / 18	85 / 22
3 vaccine doses	<1/29	6654 / 23	6 / 7	29/53	14926 / 66	225 / 58
